# Plasticity and the adaptive evolution of switchlike reaction norms under environmental change

**DOI:** 10.1093/evlett/qrad035

**Published:** 2023-08-31

**Authors:** Claudia Crowther, Stephen P Bonser, Lisa E Schwanz

**Affiliations:** Department of Integrative Biology, W.K. Kellogg Biological Station, Michigan State University, Hickory Corners, MI, United States; Evolution and Ecology Research Centre, School of Biological, Earth, and Environmental Sciences, UNSW Sydney, Sydney, NSW, Australia; Evolution and Ecology Research Centre, School of Biological, Earth, and Environmental Sciences, UNSW Sydney, Sydney, NSW, Australia

**Keywords:** adaptation, environmental sex determination, phenotypic plasticity, reaction norm, threshold trait

## Abstract

Phenotypic plasticity is often posited as an avenue for adaptation to environmental change, whereby environmental influences on phenotypes could shift trait expression toward new optimal values. Conversely, plastic trait expression may inhibit adaptation to environmental change by reducing selective pressure on ill-adapted traits. While plastic responses are often assumed to be linear, nonlinear phenotype–environment relationships are common, especially in thermally sensitive traits. Here we examine nonlinear plasticity in a trait with great ecological and evolutionary significance: sexual phenotype in species with environmental sex determination (ESD). In species with ESD, development switches between male and female at an environmental threshold (the inflection point). The inflection point is a key trait for adaptive responses to changing environments and should evolve toward the new optimum in order to maintain evolutionarily stable sex ratios. We used an individual-based theoretical model to investigate how two forms of plasticity in the ESD reaction norm—the nonlinear slope of the reaction norm and a linear shift in the inflection point—influence the evolution of the inflection point under climate warming. We found that steeper reaction norm slopes (high nonlinear plasticity) promoted evolution toward new optimal phenotypes (higher inflection points). In contrast, increased linear plasticity in the inflection point (shift) hindered adaptive evolution. Additionally, populations in moderate warming scenarios showed greater adaptive evolution of the inflection point compared with populations in extreme warming scenarios, suggesting that the proximity of existing phenotypes to new optimal phenotypes influences evolutionary outcomes. Unexpectedly, we found greater population persistence under high climate variability, due to the increased production of rare-sex individuals in unusually cold years. Our results demonstrate that different forms of phenotypic plasticity have crucially different effects on adaptive evolution. Plasticity that prevented sex ratio bias hindered the evolution of the inflection point, while plasticity that exacerbated sex ratio bias promoted adaptation to environmental change.

## Introduction

Phenotypic plasticity is prevalent across living organisms and often promotes fitness in variable environments (Agrawal, 2001; Nijhout, 2003; Sultan, 2000). However, it remains unclear whether plasticity facilitates or hinders evolutionary responses to environmental change (Connover et al., 2009; Ghalambor et al., 2007; Hendry, 2016). Adaptive plasticity that increases fitness across environments can promote population survival after environmental change and allow migration to new environments, thereby facilitating evolution (Agrawal, 2001; Chevin et al., 2010; Kelly, 2019; Lande, 2009; Scheiner et al., 2017). However, selective pressure on plastic traits may be reduced if the plastic phenotype is close to the new optimal trait value, which could hinder adaptive evolution (Huey et al., 2003; Ghalambor et al., 2007; Snell-Rood et al., 2018). Moreover, evolutionary responses to environmental change may depend critically on specific fitness consequences arising from the shape of a trait’s reaction norm (e.g., linear vs. nonlinear), which has rarely been investigated (Connover et al., 2009; Crispo, 2007; De Jong, 2005; Diamond & Martin, 2016; Ghalambor et al., 2007; Hendry, 2016; Paenke et al., 2007; Snell-Rood et al., 2018). Thus, we lack a fundamental understanding of whether plasticity facilitates or impedes evolution under environmental change and whether the shape of the reaction norm influences the response.

**Figure 1.**
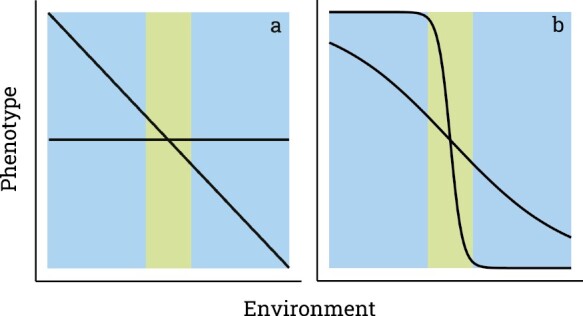
Examples of (A) linear and (B) nonlinear reaction norms of plasticity. The green-shaded area represents the environmental conditions in which the trait is normally expressed, while the blue-shaded area represents environmental change. In a linear reaction norm, the relative plasticity of a genotype compared to another is consistent across all environments, while in a nonlinear reaction norm, plasticity varies across an environmental gradient. With among-individual variation in a nonlinear reaction norm, genotypes can differ in their relative plasticity depending on the environmental range.

Traits with linear and nonlinear plasticity have differing fitness landscapes that could drive different evolutionary responses to change. In a trait with a linear reaction norm, a given change in the environment leads to a consistent change in phenotype regardless of whether the environment is average or extreme (Figure 1B). Conversely, in a nonlinear reaction norm, the same degree of change in the environment will lead to different phenotypic changes depending on the environment itself (Figure 1B). Thus, selection after environmental change is likely to be much less consistent under nonlinear plasticity than under linear plasticity. For example, many thermally sensitive traits have nonlinear reaction norms, suggesting that novel plastic expression in changing climates could produce unexpected evolutionary outcomes (Bulté & Blouin-Demers, 2006; Malusare et al., 2022; Salachan et al., 2019). Surprisingly, nonlinear reaction norms are often overlooked or simplified to linear versions across empirical and theoretical work. (Bulté & Blouin-Demers, 2006; Gibert et al., 1998).

The impact of different forms of plasticity on evolution can be examined using switchlike traits, which inherently contain both linear and nonlinear forms of plasticity within the same selective environment (Box 1). Switchlike reaction norms are typical of plastic traits that are discrete or discontinuous in distribution, characterized by at least two extreme trait states, infrequent expression of intermediate traits, and an inflection point (threshold environmental value) where trait expression switches from one extreme to the other (Box 1A). Such traits are common in nature and are often subject to frequency-dependent selection (FDS), examples include sex (male/female), alternate male morphologies, color morphs, variant leaf morphology, number of digits on a limb, eusocial castes, migration versus residency, or divorce versus mate fidelity (Charnov & Bull, 1977; Chevin & Lande, 2013; Debes et al., 2020; Germain et al., 2018; Kamakura, 2011; Moczek & Emlen, 1999; Ostrowski et al., 2000; Suzuki & Nijhout, 2006; Wells & Pigliucci, 2000; Wright, 1934). Two key features of switchlike reaction norms—the slope and the inflection point—are targets of adaptive evolutionary responses to variable environments or novel environmental change (Chevin & Lande, 2013; Hulin et al., 2009; Mitchell & Janzen, 2010; Schwanz & Proulx, 2008; Schwanz et al., 2010). Importantly, these two traits relate to plasticity that is either nonlinear (the steepness of the slope; “slope plasticity”) or linear (the ability of the inflection point to respond to the environment; “shift plasticity”; see Box 1). Despite the importance of switchlike reaction norms in nature, and their potential as a model system for investigating the evolutionary impact of different forms of plasticity, there has been limited research investigating how plasticity in discrete traits affects trait evolution in response to environmental change (Chevin & Lande, 2013; Suzuki & Nijhout, 2006).

Box 1. **Plasticity in Switchlike Traits**

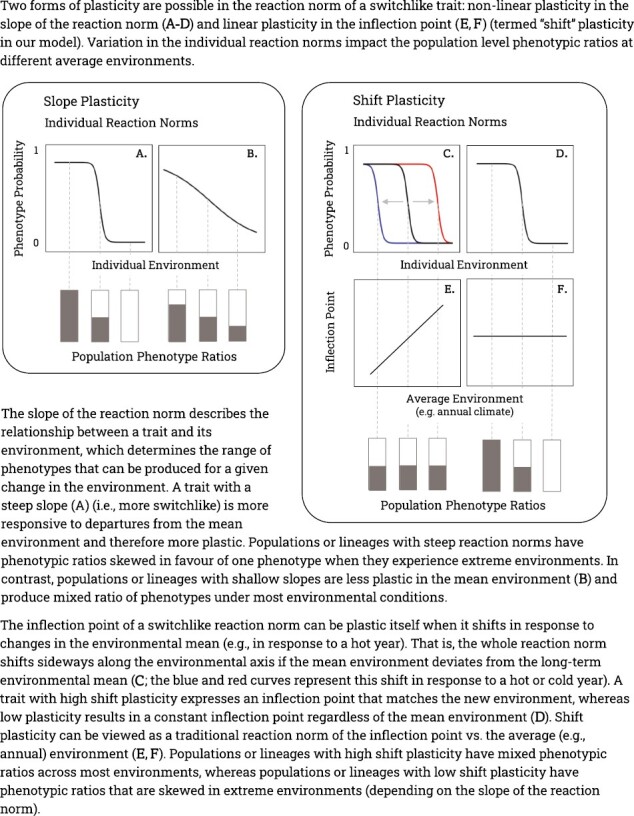

We aim to compare how two forms of plasticity in switchlike traits impact evolutionary responses to changing environments, using a fundamental, discrete trait—the sex expressed by an individual—as a model. When sex expression depends on the environment (e.g., hermaphroditism, condition-dependent offspring sex ratios, environmental sex determination [ESD]), directional change in the average environment often leads to biased sex ratios and resultant selection to equilibrate the sex ratio (Bull, 1981; Charnov & Bull, 1977; Schwanz & Georges, 2021). Responses to this selection include evolution of the inflection point of sexual phenotype to match the new environmental mean. Here we use an individual-based simulation model of ESD, to ask how nonlinear (slope) and linear (shift) forms of plasticity within switchlike reaction norms affect the evolution of the inflection point (here, the pivotal temperature). We hypothesize that selection on the sex ratio directs evolution of the pivotal temperature, whereby any plasticity that results in biased sex ratios will promote evolution of the pivotal temperature. Specifically, we predict that high slope plasticity (nonlinear) will exacerbate sex ratio biases and will therefore promote adaptive evolution of the pivotal temperature. In contrast, we expect high shift plasticity (linear) will ameliorate sex ratio biases and therefore impede adaptive evolution of the pivotal temperature.

## Methods

### Overview of plasticity scenarios

**Figure 2.**
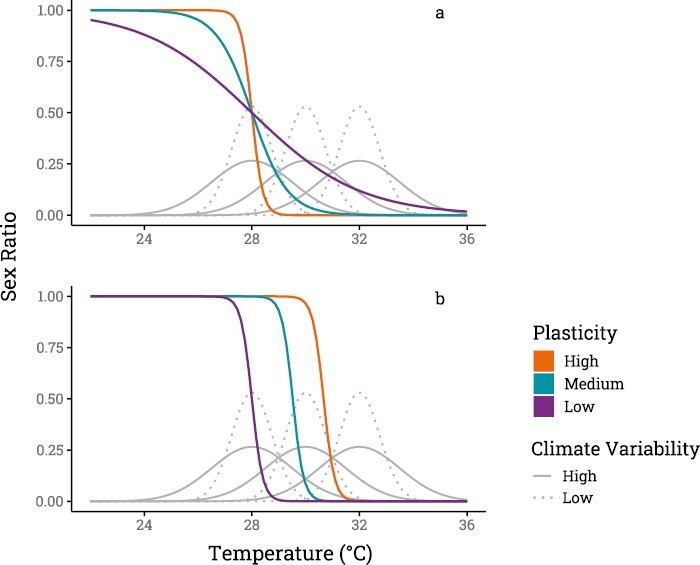
Shapes of simulated reaction norms at different levels of slope and shift plasticity. (A) Low, moderate, and high values of reaction norm slope plasticity. (B) Reaction norms produced by low, moderate, and high shift plasticity, with a *T*_*piv*_ of 28 °C in a 32 °C mean climate (see Box 1E and F for traditional reaction-norm representation of shift plasticity). Values for plasticity parameters are presented in Table 1. In both panels, the gray curves represent distributions of nest temperatures at 28 °C, 30 °C, and 32 °C with different levels of variability (high = 1.5 °C, low = 0.75 °C).

We designed an individual-based simulation model to examine how the pivotal temperature (*T*_*piv*_) of sexual development evolves in response to climatic warming in the presence of two different kinds of plasticity in the ESD reaction norm. First, we investigated the effect of plasticity in the slope of the switchlike reaction norm (“*slope*”) (Figure 2A). Second, we investigated the effect of pivotal temperature plasticity in response to annual climatic fluctuations (“*shift*”) (Figure 2B).

**Table 1.** Key variables distinguishing the simulation scenarios. Gray shading indicates that a variable could evolve under the specified scenario. When *slope* and *shift* could not evolve, they were set at the level that least balanced the sex ratios: high slope plasticity and low shift plasticity.

Scenario	*T*_*piv*_	*Slope*	*Shift*	Climate
Slope	28 °C	Low, Medium, High	Low	28 °C, 30 °C, 32 °C
Shift	28 °C	High	Low, Medium, High	28 °C, 30 °C, 32 °C

In order to examine how plasticity impacts the evolutionary response of *T*_*piv*_ under a directionally changing climate, we varied the level of plasticity in *slope* or *shift* across plasticity scenarios (Figure 2, Table 1). A highly plastic switchlike reaction norm has a very steep slope, meaning that sexual phenotype is highly dependent on the environment (within the historical range). As a result, there are fewer temperatures that could produce either a male or a female hatchling. Low plasticity is reflected in a shallow slope, meaning that the relationship between sex and environment is weaker and there is a wider range of temperatures that have a chance to produce a male or female hatchling. Thus, high plasticity described by the slope *increases* sex ratio biases in an atypical or changing climate (Box 1A, Figure 2A). High shift plasticity means that the phenotypic *T*_*piv*_ tracks more closely to annual mean nest temperatures (i.e., warmer *T*_*piv*_ in warmer years). Low shift plasticity means that the same *T*_*piv*_ being expressed regardless of annual temperature. Thus, high plasticity described by the shift *reduces* sex ratio biases in an atypical or changing climate (Box 1C, Figure 2B). In “slope scenarios,” both the *T*_*piv*_ and *slope* traits could evolve via mutation, while there was no shift plasticity or mutation (highest potential for sex ratio bias; Table 1). In “shift scenarios,” both *T*_*piv*_ and *shift* could evolve via mutation, while the *slope* was constrained at the steepest level without mutation (highest potential for sex ratio bias; Table 1). The plasticity scenario (shift/slope) and the level of plasticity (low, medium, high) were chosen randomly at the start of each replicate simulation, with approximately 25 replicates of each scenario (sample sizes are presented in Supplementary Table S1).

We explored the impact of the two types of plasticity on *T*_*piv*_ evolution across three levels of mean global climate and two levels of climate variability. We expected that the relationship between plasticity and evolution would depend on the selection pressure invoked by climatic differences and the consistency of that pressure across years. The three global temperature scenarios (*T*_*glob*_) were as follows: a base climate scenario of 28 °C, a moderate warming scenario of 30 °C, and an extreme warming scenario of 32 °C. The two climate variability scenarios were determined by the standard deviation of climate between years (SDbw): high variability (1.5 °C) and low variability (0.75 °C), selected based on natural nest temperatures in wild reptile species (based on data from four species, Schwanz et al., 2020, Supplementary Material). Global temperature and climatic variability were chosen randomly from these options at the start of each simulation replicate.

### Temperature-dependent sex determination

The simulation used a diploid genetic system, which prevents the rapid spread of extreme phenotypes and reflects the biology of species with ESD. Individuals in the population had three genes (*T*_*piv*_, *slope*, and *shift*), with two alleles at each locus. Phenotypes were expressed as the mean of both alleles. We modeled ESD as a logistic curve (Figure 2), with males developing at low temperatures and females developing at high temperatures, as in type 1a ESD (Valenzuela, 2004). We used a modified version of Schwanz and Proulx’s (2008) equation for sex determination that includes *shift*:


$$\begin{array}{l} r=\frac{1}{1+e^{-(td-(T_{piv}\;\;\;+\;\;\;shift(T_{ann}-T_{base})))slope}} \end{array}$$ (1)

where *r* is the probability of being male, *td* is the developmental temperature, *T*_*piv*_ is the pivotal temperature, *slope* is the slope of the logistic curve (slope plasticity, Figure 2A), *shift* multiplies the deviation in annual climate to adjust the pivotal temperature (shift plasticity, Figure 2B), *T*_*ann*_ is the annual climate, and *T*_*base*_ is the baseline climate (28 °C). Individual sex was determined stochastically by comparing *r* to a random number chosen from a uniform distribution between 0 and 1. Individuals with higher values of *r* were assigned male and lower values of *r* were assigned female.

### Population cycle

At the start of each replicate simulation, populations were seeded with 500 individuals that varied in developmental temperature and genetics. For these individuals, we assumed the mean developmental temperature was 28 °C regardless of which climate scenario was chosen. This was done to prevent the immediate formation of a population with no males in warm climate scenarios and accurately represents an established population that subsequently experiences climatic warming. Each individual was assigned alleles at the three ESD genes based on the chosen scenario. Alleles were drawn from a normal distribution with a mean of the chosen scenario parameter value (*slope* and *shift*) and a standard deviation of approximately 1% of the potential evolutionary range (Table 2). We assumed populations were initially adapted to the base climate scenario and assigned mean population *T*_*piv*_ equal to 28 °C, with a standard deviation that placed extreme genotypic values near the moderate warming scenario (30 °C).

**Table 2.** Description of simulation parameters. Parameters that varied across simulation scenarios are bolded.

Parameter	Description	Value(s)
Climate		
*** T***_***glob***_	**The average global temperature of a scenario.**	**28 °C, 30 °C, 32 °C**
** SD**_**bw**_	**The between-year standard deviation in annual mean temperatures.**	**0.75 °C, 1.5 °C**
* T*_*base*_	The climate that individuals are initially adapted to (mean *T*_*piv*_ = *T*_*glob*_).	28 °C
* T*_*ann*_	The annual temperature, chosen from a normal distribution with *T*_*glob*_ as the mean. and SD_bw_ as the standard deviation.	Generated randomly each year in the simulation
SD_wi_	The within-year standard deviation in developmental temperatures.	1.2
* td*	Developmental temperature of a simulated nest, chosen from a normal distribution with *T*_*ann*_ as the mean and SD_wi_ as the standard deviation.	Generated randomly each year in the simulation
Genetics		
*** Slope***	**The slope of the switchlike reaction norm.**	**−0.5, −1.5, −5**
* Slope* SD	Standard deviation of initial population variation in slope.	0.05
*** Shift***	**The ability of an individual to express a phenotypic *T***_***piv***_ **other than its genetic *T***_***piv***_ **, influenced by the deviation of *T***_***ann***_ **from *T***_***base***_.	**0, 0.38, 0.6666**
* Shift* SD	Standard deviation of initial population variation in *shift*.	0.01
* T*_*piv*_	The initial temperature with an equal chance of producing a male or female hatchling.	28 °C
* T*_*piv*_ SD	The standard deviation of initial population variation in *T*_*piv*_.	0.5
Mutation rate	The percentage of hatchlings that receive mutations.	2%
Mutation range (Mutvar_array)	The standard deviation of mutation possible for *T*_*piv*_, *shift* and *slope*.	0.08, 0.1, 0.02
Demographics		
Ni	The initial population size of each replicate simulation.	500
T	The maximum number of iterations (years) in each simulation.	50,000
Mortality (Ad_mort)	The proportion of adults that leave the population each year.	0.1
* Phi*	A constant in the female fertility equation.	2
* F*_*scale*_	Scale for female fecundity.	0.02
const	Density dependant constant for first-year survival.	0.01
* M*_*lim*_	Expression of male limitation on female fertility.	0.01

Each year (iteration) the annual mean temperature (*T*_*ann*_) was drawn from a normal distribution with *T*_*glob*_ as the mean and SDbw as the standard deviation. When females reproduced, each nest temperature was randomly chosen from a normal distribution with a mean equal to the annual temperature and a standard deviation of SD_wi_ (1.2 °C), which reflects average among-nest variation in wild reptile nests (Schwanz et al., 2020). These nest temperatures became the developmental temperature (*td*) of the offspring from that nest. It is important to note that our simulation does not model plasticity in maternal nesting behavior.

Mating occurred in each iteration. Male mates were chosen randomly with replacement for each adult female. Clutch size (CS) was exponentially related to maternal developmental temperature in order to establish sex-differential fitness as a function of temperature and, therefore, selection for ESD. This reflects the likely selective pressures that result in the evolution of ESD (Charnov & Bull, 1977). Because climatic warming and FDS on sex can lead to selection for the loss of ESD, we wanted to include the selective pressures that promote the maintenance of ESD in wild populations. Clutch size was determined by the following equation:


$$\begin{array}{l} CS\;\;\;=\;\;\;(F_{scale}{\left(td\right)}^{Phi})\left(\frac{OSR}{OSR+M_{lim}}\right) \end{array}$$ (2)

The fecundity scaler (*F*_*scale*_) was multiplied by the female incubation temperature (*td*) to the power of *Phi*, so that the CS ranged from 10 to 25 over temperatures 22−35 °C. In our simulation, female fertility was also limited by the number of males present in the population. We used the male limitation equation from Rankin and Kokko (2007). The *OSR* is the operational sex ratio, and *M*_*lim*_ represents the impact that male population proportion has on CS. We chose *M*_*lim*_ = 0.01 to ensure males only limited female fecundity when the proportion of males was below 10%. This value prevents the unrealistic continuation of a population with very low males, and the rapid evolution of a population by the spread of genes from a few unusual males.

New offspring received one allele from each parent for their *T*_*piv*_, *slope*, and *shift*, chosen randomly from each parent’s allelic complement. Offspring had a 2% chance to become a mutant. Mutants experienced mutations on all alleles at each locus that was assigned to mutate based on the scenario. Mutant allelic values were chosen randomly from a normal distribution with a mean equal to the original allelic value and a standard deviation of 2% of the biologically realistic range for the trait (Table 2).

Adult mortality occurred at a fixed rate (10%) after breeding. Juvenile mortality was density dependent, given by the following equation:


$$\begin{array}{l} Mort=\;1-e^{\left(-const(n_{Adults})\right)} \end{array}$$ (3)

where *Mort* is the probability of mortality, *const* is a density-dependent constant with the value 0.01, and *n*_*Adults*_ is the number of adults alive in the iteration. Surviving juveniles were recruited to the adult population at the end of each iteration. That is, animals matured at 1 year of age.

### Model validation and sensitivity analysis

Across our scenarios, we hypothesized that we would have simultaneous selection for higher *T*_*piv*_ (driven by warmer climates), higher slope plasticity (driven by sex-differential fertility/fecundity), and higher shift plasticity (driven by annual fluctuations in climate and sex ratios; e.g., Schwanz & Proulx, 2008; Schwanz et al., 2010). Because our scenarios examined concomitant evolution of *T*_*piv*_ and plasticity (*shift*/*slope*), we briefly examined the evolution of each trait independently (without mutation in the other traits) to validate our assumptions (see Supplementary Table S2). We confirmed that the pivotal temperature evolved to near-perfectly match the warmed climatic mean (30 °C and 32 °C; *slope* fixed at “high,” *shift* fixed at “low,” Supplementary Figure S1). We validated that a sex-differential link between fitness and temperature drives ESD toward appreciably negative slopes under the base climate (*shift* fixed at “low,” Supplementary Figure S2). Finally, we found that there was a threshold starting *shift* at ~0.4 that facilitated the evolution of very high *shift* values (>0.8, Supplementary Figure S3). Additionally, we performed analyses to assess sensitivity of the simulation results to mutation range (Supplementary Figure S4, Supplementary Tables S3 and S4) and adult mortality rate (Supplementary Figure S5, Supplementary Tables S5 and S6).

The simulation was conducted in MATLAB (2021), with the Statistics and Machine Learning Toolbox add-on. Graphs were constructed in RStudio using the Tidyverse and Patchwork packages (Pedersen, 2020; R Core Team, 2021; Wickham et al., 2019).

## Results

### Persistence

**Figure 3.**
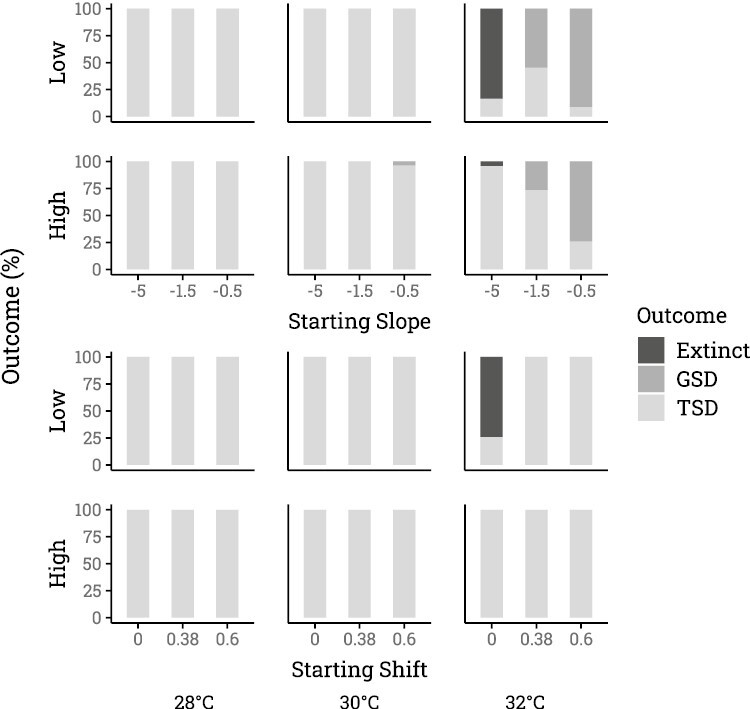
Outcome for replicates in all scenarios, including replicates that went extinct, replicates that transitioned to GSD and replicates that maintained TSD. The top six panels show results from scenarios where slope plasticity could evolve, and the bottom six panels show results from scenarios where shift plasticity could evolve. The climate variability of the scenarios is shown on the left side of the graph at the start of each row (high = 1.5 °C, low = 0.75 °C). Extinct populations became 100% female before 50,000 generations (the length of the simulation). A reaction norm slope more positive than −0.1 was classified as a switch to genetic sex determination (GSD). Sample sizes for each scenario are presented in Supplementary Table S1.

The majority of replicates (95.4%) survived for the full duration of the simulation (Figure 3). Most replicates resulting in extinction came from *slope* and *shift* scenarios in the hottest (32 °C) and least variable climates with the least buffering of sex ratios (initial *slope* = −5 and *shift* = 0). These are the parameter combinations that cause the greatest sex ratio biases. Higher *shift* and lower *slope* values promoted population survival by buffering sex ratios (Figure 3).

In some populations where *slope* was allowed to evolve, *slope* became so shallow as to constitute a loss of ESD (defined as *slope* ≥ −0.1) (Figure 3, top six panels). This occurred primarily in hot climates (32 °C) with medium or low slope plasticity.

### Changes in pivotal temperature over time

**Figure 4.**
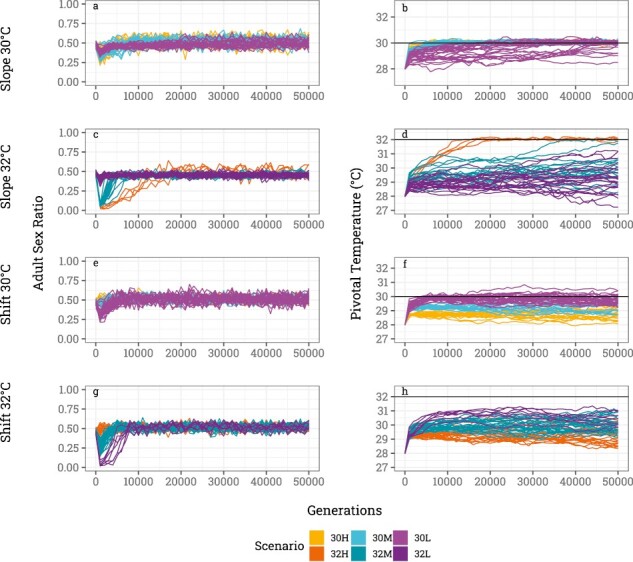
The adult sex ratios and mean pivotal temperatures (*T*_*piv*_) for populations in replicate simulations over time, at different levels of climate and starting plasticity. In scenarios labeled “shift,” shift plasticity and *T*_*piv*_ can evolve, while slope plasticity is fixed. In scenarios labeled “slope,” slope plasticity and *T*_*piv*_ can evolve, while shift plasticity is fixed. Only results from warmed climate scenarios are presented as there was little change in *T*_*piv*_ or adult sex ratio in baseline 28 °C climate populations. A ratio of 1 is 100% male, a ratio of 0 is 100% female. Starting levels of plasticity in the scenario key are as follows: H = High, M = Moderate, L = Low. Results shown are from low climate variability treatments.

When the slope of the ESD reaction norm was allowed to evolve, there was an interaction between climate and plasticity that affected the speed of pivotal temperature evolution. With modest warming (30 °C scenarios), populations with high and medium plasticity (steeper slopes) evolved to match the new climate (final mean *T*_*piv*_ = 30° C; Figure 4B, orange and blue) much quicker than populations with low plasticity (shallow slope, purple). In contrast, in hot climates (32 °C scenarios), the few high plasticity populations that survived reached a mean pivotal temperature of 32 °C after substantial delay (Figure 4D, orange), while populations with medium and shallow slopes experienced little evolution of the pivotal temperature at all (Figure 4D, blue and purple).

The starting level of shift plasticity had a less pronounced effect on the speed of *T*_*piv*_ evolution, with the results being somewhat reversed from slope plasticity (Figure 4F and H). Across both climate scenarios, it was the low plasticity scenarios (*shift* = 0, purple) where *T*_*piv*_ continued to increase for a longer period of time than in the medium or high plasticity scenarios (blue and orange), with the latter scenarios plateauing at around the same time but at lower values of *T*_*piv*_ (Figure 4F and H). In all plasticity scenarios, *T*_*piv*_ evolution followed nearly identical patterns under high and low climate variability (Supplementary Figure S6).

### Changes in sex ratios over time

The impacts of climate and plasticity on adult sex ratios mirrored the patterns observed for *T*_*piv*_ evolution. Under mild warming (30 °C) and when *slope* was allowed to evolve, sex ratios were initially female biased, but quickly increased to parity (Figure 4A). In contrast, under extreme warming (32 °C), steeper initial slopes (medium and high slope plasticity) led to strongly female-skewed sex ratios initially, with a substantial delay to reach parity, particularly in the high plasticity runs where a 50:50 sex ratio appears coincident with *T*_*piv*_ reaching 32 °C (Figure 4C).

A similar pattern occurred when *shift* was allowed to evolve, reversed with respect to plasticity level (Figure 4E and G). Under mild warming (30 °C), sex ratios quickly reached a stable trajectory between 40% and 60% male. Under extreme warming (32 °C), low plasticity populations (initial *shift* = 0) were strongly female skewed for a greater period of time than populations in medium or high plasticity scenarios. Across all scenarios, higher climate variability increased fluctuations in sex ratios on a per-cohort basis, though the directional patterns through time remained similar (Supplementary Figure S6e and g).

### Impact of slope plasticity on final *T*_*piv*_

**Figure 5.**
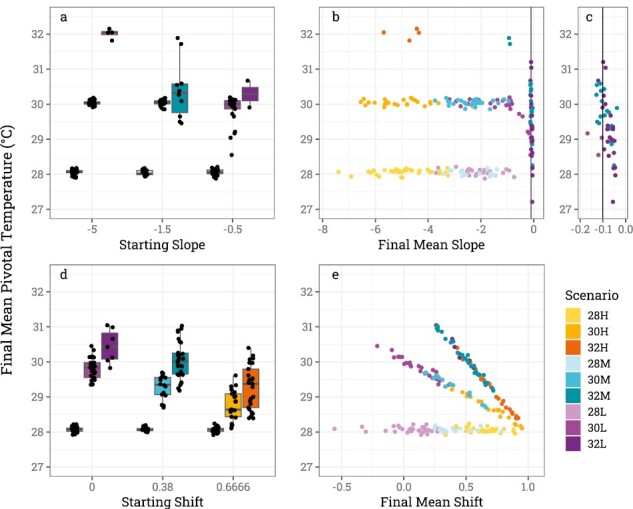
The final mean pivotal temperature of replicate simulations over starting and final mean plasticity levels (mean of the last 1,000 generations). Levels of plasticity in the scenario key are as follows: H = High, M = Moderate, L = Low. Results shown are from low climate variability treatments. (A) Final mean pivotal temperatures for the three levels of starting *slope* plasticity in all climate scenarios. Only replicates that maintained ESD are included in the boxplots. (B) Final mean pivotal temperatures and final mean slopes of replicates in slope plasticity scenarios, the black line indicates the boundary between ESD and a GSD-like slope. (C) An expansion of (B), centered around the black line. It shows the scenarios that lost ESD (slope > −0.1) on the right side of the black line. (D) Final mean pivotal temperatures for the three levels of starting shift plasticity in all climate scenarios. (E) Final mean pivotal temperatures and final mean shifts of replicates in shift plasticity scenarios.

Under baseline (28 °C) and mild warming (30 °C) scenarios, the three levels of *slope* all produced final mean *T*_*piv*_ roughly equaling the global climate (Figure 5A and B). In these scenarios, the slope generally stayed steep (for high, orange) or became steeper (for medium, blue, and low, purple). The exception is for some mild warming (30 °C), shallow slope populations, where the slope became shallower and *T*_*piv*_ did not reach 30 °C. In contrast, in the 32 °C scenarios, high slope plasticity promoted *T*_*piv*_ evolution (Figure 5A and B). Replicates with high plasticity (steep slopes) evolved pivotal temperatures closer to 32 °C (if they did not go extinct) compared with the replicates with shallower starting slopes where the slope simply evolved to be very shallow (Figure 5B and C, orange vs. purple and blue). This pattern was also the same in the high variability climates, where most of the high slope replicates persisted. With high climate variability scenarios, however, a greater proportion of the medium slope replicates retained ESD-like slopes and had final pivotal temperatures near 32 °C (Supplementary Figure S7).

### Impact of shift plasticity on final *T*_*piv*_

In contrast to the positive relationship between slope plasticity and *T*_*piv*_ evolution, we found a negative relationship between the level of shift plasticity and evolution of the pivotal temperature. Across both warmed climate scenarios (30 °C or 32 °C), increased shift plasticity reduced the final extent of *T*_*piv*_ evolution (Figure 5D and E).

Additionally, global climate had an effect on *T*_*piv*_ evolution. As is to be expected, replicates evolved at warmer pivotal temperatures in warmer global climates. In both warming climates, lower plasticity runs showed greater increases in pivotal temperature during the course of the simulation (Figure 5D). Climate variability appears to have a negligible impact on *T*_*piv*_ evolution when interacting with shift plasticity.

### Sensitivity to mutation and adult mortality

A higher mutation rate was associated with increased *T*_*piv*_ evolution in low plasticity scenarios for both slope and shift plasticity (see Supplementary Figure S4). Increased mortality rate (shorter life spans) increased *T*_*piv*_ evolution when the initial slope was shallow (low slope plasticity), with *T*_*piv*_ reaching the climatic mean and ESD being maintained. In contrast, increased mortality decreased *T*_*piv*_ evolution for low shift plasticity scenarios because high *shift* values evolved, precluding the need for *T*_*piv*_ evolution.

## Discussion

We aimed to determine how trait evolution in response to environmental change is affected by different forms of plasticity (linear vs. nonlinear reaction norms). We examined switchlike reaction norms associated with ESD, which contain both nonlinear plasticity in the slope of the reaction norm between sex and the environment and linear plasticity in the pivotal temperature (“shift plasticity”). We found that slope and shift plasticity have opposite effects on the evolution of the pivotal temperature, which is a consequence of the different ways that nonlinear and linear reaction norms influence fitness. Steep slopes, which indicate a highly plastic relationship between sex (male/female) and the baseline environment, promoted the evolution of the pivotal temperature toward the new mean temperature of the environment (Figure 5A and B). In contrast, high plasticity in the pivotal temperature itself (shift) hindered the pivotal temperature evolution (Figure 5D and E). Additionally, it was easier for the pivotal temperature to reach the new mean temperature in the moderately warmed environment compared to the extreme warming scenario, as starting populations contained some alleles that were suited to this environment. Our investigation demonstrates that the strength of plasticity and the shape of the reaction norm dictate when and how selection acts on switchlike traits.

The evolution of the pivotal temperature proceeded in vastly different ways depending on the form of plasticity examined. Both high slope plasticity and low shift plasticity were associated with female-biased population sex ratios after climatic warming. Because there is strong selection operating on sex ratios, alleles for producing the rare sex at the new mean are highly favored in these biased populations (Bull, 1981; Charnov & Bull, 1977; Chevin et al., 2021; Schwanz & Georges, 2021). Thus, the two forms of plasticity have opposite effects on the evolution of the pivotal temperature because they have opposite effects on sex ratio biases. However, this explanation overlooks the crucial impact of nonlinearity in the switchlike reaction norm: populations that were initiated with steep slopes (high plasticity) within the historical environmental range actually have lower plasticity in sexual outcome in a warmed climate than lineages initiated with shallower slopes in the historical environment (Figure 1A). So, similar to shift plasticity, higher slope plasticity measured in the novel environment reduces selection on the pivotal temperature (via reduced sex ratio biases) and its subsequent evolutionary response. Overall, our results indicate that adaptive evolution is promoted when traits are exposed to strong selection in a new environment, which occurs with traits that are not plastic or have nonlinear plasticity. Our findings add nuance to the debate regarding the effect of plasticity on evolution by demonstrating that adaptive plasticity can promote or hinder evolution, depending on the shape of the reaction norm and the environmental range across which plasticity is measured (Connover et al., 2009; Crispo, 2007; De Jong, 2005; Diamond & Martin, 2016; Ghalambor et al., 2007; Hendry, 2016; Huey & Kingsolver, 1993; Huey et al., 2003; Paenke et al., 2007; Snell-Rood et al., 2018; Sultan, 1987, 1995, 2000).

The coevolution of traits in our model highlighted that there are several means by which populations can regain an evolutionarily stable state under climate warming. Shallow slopes, high shifts, and the evolution of inflection points represented alternative solutions to the problem of ill-adapted phenotypes (here, the overproduction of the common sex). The evolutionary outcome was a compensatory surface across trait values. For shift, there was a negative linear relationship between the final pivotal temperature and the final shift level that evolved—where *shift* increased from starting levels, pivotal temperature evolved to a lesser extent and vice versa. For slope plasticity, the trade-off between plasticity and pivotal temperature evolution was nonlinear. Specifically, pivotal temperature evolved to the new climatic mean equally for all final slopes up until a shallow slope threshold of ~−0.1 when pivotal temperature evolution seemed to drift, having lost its relevance in very shallow reaction norms (approximating genotypic sex determination, Figure 5B and C). For organisms with shallow starting slopes in extreme environments, relaxing the relationship between sex and temperature and effectively losing ESD was more evolutionarily accessible than evolving a higher pivotal temperature. These evolutionary outcomes reflect the differing fitness landscapes of traits with linear and nonlinear plasticity. The direction of selection on plasticity was consistent across starting levels of *shift* (selection for increased plasticity). In contrast, there was directional selection for slope plasticity, reduced plasticity was selected at shallow slopes, and increased plasticity was favored at steep slopes. This suggests that in a trait with a nonlinear relationship to the environment, a fitness valley, such as the barrier to evolving a steep slope from a shallow slope, is more likely than in a trait with a linear relationship to the environment. It is possible that these results are related to FDS on sex ratios, and while many switchlike traits are subject to FDS, it is not universal to traits with nonlinear reaction norms. Whether these findings are applicable to any situation where selection can act in or against the direction of plastic expression is a worthwhile question for future exploration.

The possibility of adapting to a new environment depends significantly on the distance between the possible phenotypes present in a population and the new optimal phenotype. Accordingly, in our study, the magnitude of climate warming had a significant effect on pivotal temperature evolution. Initial genetic variation included extreme pivotal temperature alleles near 30 °C, appropriate to the moderate warming climate, but not to the extreme warming climate. It is possible that our choice to model an abrupt rather than gradual increase in temperature may influence whether populations evolved a mean pivotal temperature equal to the new environmental mean, as gradual change is likely to promote population persistence. However, modeling gradual change requires making assumptions about the relative speed of new allele generation with respect to the speed of climatic warming, which would result in findings that were specific to the life history and environment of our modeled organism (Chevin et al., 2010).

In contrast to the predictable response to the extent of climatic warming, climate variability had unexpected effects on population persistence. Population extinctions almost exclusively occurred in low climate variability scenarios (Figure 3). Population extinction under low climate variability is contrary to a wealth of literature suggesting that extinctions are more likely in highly variable climates (Boyce et al., 2006; McLaughlin et al., 2002; Mustin et al., 2013; Vincenzi, 2014). However, our finding is likely to be specific to phenotypes subject to negative FDS, as climates with high variability have occasional extreme years that allow development of the rare sex (males), thereby enabling reproduction. While a steep slope and low shift promoted the evolution of the pivotal temperature, these scenarios had a high rate of extinction. In an ecological context, steeper reaction norms are associated with more biased sex ratios than shallow reaction norms and are predicted to make populations vulnerable to extinction under extreme environmental change (Hulin et al., 2009; Mitchell & Janzen, 2010). Thus, populations with steep slopes and no shift plasticity must have substantial evolvability of the pivotal temperature to avoid rapid extinction. In the context of species with ESD facing climate change, understanding heritability of the pivotal temperature and alternative forms of response to climate change (e.g., oviposition site choice) is imperative.

We have shown that plastic responses of species with ESD can greatly affect the evolutionary trajectory of ESD reaction norms and that these effects depend considerably on the type and strength of plasticity in operation. Additionally, the degree of warming and available phenotypic variation are likely to impact long-term evolutionary responses to climate change. When the climate warms and species are pushed to the extreme ends of their tolerable thermal range, we can expect plasticity to become less linear, as traits are expressed in novel environments in which they have not previously been exposed to selection. Extinction or evolutionary loss of ESD are possibilities for ESD species under these conditions, and the ESD reaction norm slope and the presence of plasticity in the pivotal temperature are likely to influence the outcome.

## Supplementary Material

qrad035_suppl_Supplementary_Material

## Data Availability

Data are available in the Dryad repository: https://doi.org/10.5061/dryad.c2fqz61fj.

## References

[CIT0001] Agrawal, A. A. (2001). Phenotypic plasticity in the interactions and evolution of species. Science, 294(5541), 321–326. 10.1126/science.1060701 11598291

[CIT0002] Boyce, M. S., Haridas, C. V., Lee, C. T.; The NCEAS Stochastic Demography Working Group. (2006). Demography in an increasingly variable world. Trends in Ecology and Evolution, 21(3), 141–148. 10.1016/j.tree.2005.11.018 16701490

[CIT0003] Bull, J. J. (1981). Sex ratio evolution when fitness varies. Heredity, 46(1), 9–26. 10.1038/hdy.1981.2

[CIT0004] Bulté, G., & Blouin-Demers, G. (2006). Cautionary notes on the descriptive analysis of performance curves in reptiles. Journal of Thermal Biology, 31, 287–291.

[CIT0005] Charnov, E. L., & Bull, J. J. (1977). When is sex environmentally determined? Nature, 266(5605), 828–830. 10.1038/266828a0 865602

[CIT0006] Chevin, L. -M., Gompert, Z., & Nosil, P. (2021). Frequency dependence and the predictability of evolution in a changing environment. Evolution Letters, 6(1), 21–33. 10.1002/evl3.266 35127135 PMC8802243

[CIT0007] Chevin, L. -M., & Lande, R. (2013). Evolution of discrete phenotypes from continuous norms of reaction. American Naturalist, 182(1), 13–27. 10.1086/670613 23778223

[CIT0008] Chevin, L. -M., Lande, R., & Mace, G. M. (2010). Adaptation, plasticity, and extinction in a changing environment: Towards a predictive theory. PLoS Biology, 8, e1000357. 20463950 10.1371/journal.pbio.1000357 PMC2864732

[CIT0009] Connover, D. O., Duffy, T. A., & Hice, L. A. (2009). The covariance between genetic and environmental influences across ecological gradients: Reassessing the evolutionary significance of Countergradient and Cogradient variation. Annals of the New York Academy of Sciences, 1168, 100–129. 19566705 10.1111/j.1749-6632.2009.04575.x

[CIT0010] Crispo, E. (2007). The Baldwin effect and genetic assimilation: Revisiting two mechanisms of evolutionary change mediated by phenotypic plasticity. Evolution, 61(11), 2469–2479. 10.1111/j.1558-5646.2007.00203.x 17714500

[CIT0011] de Jong, G. (2005). Evolution of phenotypic plasticity: Patterns of plasticity and the emergence of ecotypes. New Phytologist, 166(1), 101–117. 10.1111/j.1469-8137.2005.01322.x 15760355

[CIT0012] Debes, P. V., Piavchenko, N., Erkinaro, J., & Primmer, C. R. (2020). Genetic growth potential, rather than phenotypic size, predicts migration phenotype in Atlantic salmon. Proceedings of the Royal Society B: Biological Sciences, 287(1931), 20200867. 10.1098/rspb.2020.0867 PMC7423655 32693717

[CIT0013] Diamond, S. E., & Martin, R. A. (2016). The interplay between plasticity and evolution in response to human-induced environmental change. F1000, 5, 2835. 10.12688/f1000research.9731.1 PMC5147521 28003883

[CIT0014] Germain, R. R., Wolak, M. E., & Reid, J. M. (2018). Individual repeatability and heritability of divorce in a wild population. Biology Letters, 14(6), 20180061. 10.1098/rsbl.2018.0061 29899127 PMC6030595

[CIT0015] Ghalambor, C. K., McKay, J. K., Carroll, S. P., & Reznick, D. N. (2007). Adaptive vs non-adaptive phenotypic plasticity and the potential for contemporary adaptation in new environments. Functional Ecology, 21, 394–407.

[CIT0016] Gibert, P., Moreteau, B., David, J. R., & Scheiner, S. M. (1998). Describing the evolution of reaction norm shape: Body pigmentation in *Drosophila*. Evolution, 52(5), 1501–1506. 10.1111/j.1558-5646.1998.tb02032.x 28565397

[CIT0017] Hendry, A. P. (2016). Key questions on the role of phenotypic plasticity in eco-evolutionary dynamics. Heredity, 107(1), 25–41. 10.1093/jhered/esv060 26297912

[CIT0018] Huey, R. B., Hertz, P. E., & Sinervo, B. (2003). Behavioral drive versus behavioral inertia in evolution: A null model approach. American Naturalist, 161(3), 357–366. 10.1086/346135 12699218

[CIT0019] Huey, R. B., & Kingsolver, J. G. (1993). Evolution of resistance to high temperature in ectotherms. American Naturalist, 142, S21–S46.

[CIT0020] Hulin, V., Delmas, V., Girondot, M., Godfrey, M. H., & Guillon, J. -M. (2009). Temperature-dependent sex determination and global change: Are some species at greater risk? Oecologia, 160(3), 493–506. 10.1007/s00442-009-1313-1 19277719

[CIT0021] Kamakura, M. (2011). Royalactin induces queen differentiation in honeybees. Nature, 473(7348), 478–483. 10.1038/nature10093 21516106

[CIT0022] Kelly, M. (2019). Adaptation to climate change through genetic accommodation and assimilation of plastic phenotypes. Philosophical Transaction of the Royal Society B: Biological Science, 374(1768), 20180176. 10.1098/rstb.2018.0176 PMC6365860 30966963

[CIT0023] Lande, R. (2009). Adaptation to an extraordinary environment by evolution of phenotypic plasticity and genetic assimilation. Journal of Evolutionary Biology, 22(7), 1435–1446. 10.1111/j.1420-9101.2009.01754.x 19467134

[CIT0024] Malusare, S. P., Zilio, G., & Fronhofer, E. A. (2022). Evolution of thermal performance curves: A meta-analysis of selection experiments. Journal of Evolutionary Biology, 36(1), 15–28. 10.1111/jeb.14087 36129955 PMC10087336

[CIT0025] MATLAB. (2021). version 9.10 (R2021a). The MathWorks Inc.

[CIT0026] McLaughlin, J. F., Hellmann, J. J., Boggs, C. L., & Ehrlich, P. R. (2002). Climate change hastens population extinctions. Proceedings of the National Academy of Sciences of the United States of America, 99(9), 6070–6074. 10.1073/pnas.052131199 11972020 PMC122903

[CIT0027] Mitchell, N. J., & Janzen, F. J. (2010). Temperature-dependant sex determination and contemporary climate change. Sexual Development, 4(1–2), 192–140. 10.1159/000282494 20145383

[CIT0028] Moczek, A. P., & Emlen, D. J. (1999). Proximate determination of male horn dimorphism in the beetle *Onthophagus taurus* (Coleoptera: Scarabaeidae). Journal of Evolutionary Biology, 12, 27–37.

[CIT0029] Mustin, K., Dytham, C., Benton, T. G., & Travis, J. M. J. (2013). Red noise increases extinction risk during rapid climate change. Diversity and Distributions, 19, 815–824.

[CIT0030] Nijhout, H. F. (2003). Development and evolution of adaptive polyphenisms. Evolution and Development, 5(1), 9–18. 10.1046/j.1525-142x.2003.03003.x 12492404

[CIT0031] Ostrowski, M. -F., Jarne, P., & David, P. (2000). Quantitative genetics of sexual plasticity: The environmental threshold model and genotype-by environment interaction for phallus development in the snail *Bulinus truncatus*. Evolution, 54(5), 1614–1625. 10.1111/j.0014-3820.2000.tb00706.x 11108589

[CIT0032] Paenke, I., Sendhoff, B., & Kawecki, T. J. (2007). Influence of plasticity and learning on evolution under directional selection. American Naturalist, 170, 47–58. 10.1086/518952 17874367

[CIT0033] Pedersen, T. L. (2020). *patchwork: The Composer of Plots. R package version 1.1.1*. https://CRAN.R-project.org/package=patchwork.

[CIT0034] R Core Team. (2021). R: A language and environment for statistical computing. R Foundation for Statistical Computing. https://www.R-project.org/.

[CIT0035] Rankin, D. J., & Kokko, H. (2007). Do males matter? The role of males in population dynamics. Oikos, 116, 335–348.

[CIT0036] Salachan, P. V., Burgaud, H., & Sørensen, J. G. (2019). Testing the thermal limits: Non-linear reaction norms drive disparate thermal acclimation responses in Drosophila melanogaster. Journal of Insect Physiology, 118, 103946. 10.1016/j.jinsphys.2019.103946 31525352

[CIT0037] Scheiner, S. M., Barfield, M., & Holt, R. D. (2017). The genetics of phenotypic plasticity. XV. Genetic assimilation, the Baldwin effect, and evolutionary rescue. Ecology and Evolution, 7(21), 8788–8803. 10.1002/ece3.3429 29152178 PMC5677470

[CIT0038] Schwanz, L. E., & Georges, A. (2021). Sexual development and the environment: Conclusions from 40 years of theory. Sexual Development, 15(1–3), 7–22. 10.1159/000515221 34130303

[CIT0039] Schwanz, L. E., Georges, A., Holleley, C. E., & Sarre, S. D. (2020). Climate change, sex reversal and lability of sex-determining systems. Journal of Evolutionary Biology, 33(3), 270–281. 31951035 10.1111/jeb.13587

[CIT0040] Schwanz, L. E., Janzen, F. J., & Proulx, S. R. (2010). Sex allocation based on relative and absolute condition. Evolution, 64(5), 1331–1345. 10.1111/j.1558-5646.2009.00916.x 20002168

[CIT0041] Schwanz, L. E., & Proulx, S. R. (2008). Mutual information reveals variation in temperature-dependent sex determination in response to environmental fluctuation, lifespan and selection. Proceedings of the Royal Society of London B: Biological Sciences, 275(1650), 2441–2448. 10.1098/rspb.2008.0427 PMC2603192 18647722

[CIT0042] Snell-Rood, E. C., Kobiela, M. E., Sikkink, K. L., & Shephard, A. M. (2018). Mechanisms of plastic rescue in novel environments. Annual Review of Ecology, Evolution, and Systematics, 49, 331–354.

[CIT0043] Sultan, S. (2000). Phenotypic plasticity for plant development, function and life history. Trends in Plant Science, 5, 537–542. 11120476 10.1016/s1360-1385(00)01797-0

[CIT0044] Sultan, S. E. (1987). Evolutionary implications of phenotypic plasticity in plants. Evolutionary Biology, 21, 127–178. 10.1007/978-1-4615-6986-2_7

[CIT0045] Sultan, S. E. (1995). Phenotypic plasticity and plant adaptation. Acta Botanica Neerlandica, 44, 363–383.

[CIT0046] Suzuki, Y., & Nijhout, H. F. (2006). Evolution of a polyphenism by genetic accommodation. Science, 311(5761), 650–652. 10.1126/science.1118888 16456077

[CIT0047] Valenzuela, N. (2004). Introduction. In N. Valenzuela & V. Lance (Eds.), Temperature-dependant sex determination in vertebrates. Smithsonian Institution.

[CIT0048] Vincenzi, S. (2014). Extinction risk and eco-evolutionary dynamics in a variable environment with increasing frequency of extreme events. Journal of the Royal Society Interface, 11(97), 20140441. 10.1098/rsif.2014.0441 24920116 PMC4208378

[CIT0049] Wells, C. L., & Pigliucci, M. (2000). Heterophylly in aquatic plants: Considering the evidence for adaptive plasticity. Perspectives in Plant Ecology Evolution and Systematics, 3(1), 1–18. 10.1078/1433-8319-00001

[CIT0050] Wickham, H., Averick, M., Bryan, J., Chang, W., D’Agostino McGowan, L., François, R., Grolemund, G., Hayes, A., Henry, L., Hester, J., Kuhn, M., Pedersen, T. L., Miller, E., Bache, S. M., Müller, K., Ooms, J., Robinson, D., Seidel, D. P., Spinu, V., …Yutani, H. (2019). Welcome to the tidyverse. Journal of Open Source Software, 4, 1686.

[CIT0051] Wright, S. (1934). The results of crosses between inbred strains of guinea pigs, differing in number of digits. Genetics, 19(6), 537–551. 10.1093/genetics/19.6.537 17246736 PMC1208512

