# Supplementary material for: Plasticity and the adaptive evolution of switchlike reaction norms under environmental change
Source: Evol Lett. 2023 Aug 31;8(1):64–75. doi: 10.1093/evlett/qrad035 (PMC10872057; doi:10.1093/evlett/qrad035)
Supplement: qrad035_suppl_Supplementary_Material [file qrad035_suppl_supplementary_material.pdf]

Supplement: Plasticity and the adaptive evolution of switchlike reaction norms under environmental change

Sample Sizes for ESD Plasticity Model

Table S1. Replicate sample sizes for the 36 different scenarios in the ESD plasticity model. In all scenarios, T_{piv} could evolve. In 'slope scenarios', the slope could evolve, and in 'shift scenarios', shift could evolve.

**Plasticity Scenario**	**Plasticity Level**	**Global Temperature**	**Climate Variation**	**n**
Slope	Low	28°C	Low	26
			High	33
		30°C	Low	25
			High	27
		32°C	Low	22
			High	27
	Medium	28°C	Low	17
			High	26
		30°C	Low	28
			High	22
		32°C	Low	22
			High	19
	High	28°C	Low	36
			High	21
		30°C	Low	25
			High	23
		32°C	Low	24
			High	25
Shift	Low	28°C	Low	19
			High	28
		30°C	Low	22
			High	31
		32°C	Low	25
			High	34
	Medium	28°C	Low	20
			High	29
		30°C	Low	23
			High	32
		32°C	Low	26
			High	35
	High	28°C	Low	21
			High	30
		30°C	Low	24
			High	33
		32°C	Low	27
			High	36
**Total**				**900**

Model Validation and Sensitivity Analysis

This supplementary document contains information about the sensitivity of our model to the most relevant parameters. Test conducted are summarized in Table S1. Our sensitivity analysis required data from runs that completed 50000 generations. In general, scenarios were run only five times to gather the presented data, however scenarios with a high likelihood of extinction were run until five surviving runs were produced. When this has occurred, we have indicated it next to the results.

Table S2. Validation tests and sensitivity analyses

Test	Parameter description	Validation / Sensitivity Levels	Main Simulation level
Pivotal temperature evolution	Validation of T_{piv} evolvability at control plasticity levels. Values of shift and slope with the least moderating effect on sex ratios were chosen as the control levels.	Climate: 30°C, 32°C	Same
		Variability: 0.75°C, 1.5°C	
Slope evolution	Validation of slope evolution with different starting slopes.	-1, -2, -3, -4, -5, -6, -7	-5, -1.5, -0.5
Shift evolution	Sensitivity of shift evolution to different starting levels of shift.	0.2, 0.4, 0.6, 0.8, 1	0, 0.38, 0.6666
Mutation range	The standard deviation of new mutational values possible for a trait, normally distributed around the initial trait mean. Expressed here as a percentage of the possible initial values of T_{piv}, slope, and shift in the simulation	1%, 3%	2%
Mortality rate	The rate at which adults leave the population.	0.02, 0.5	0.1

Pivotal Temperature Evolution

When slope and T_{piv} shift were maintained at levels which had the least moderating effect on sex ratios (control values for the simulation), T_{piv} evolved to a final level close to the new mean global temperature (Fig. S1). T_{piv} evolution was not sensitive to temperature variability; however, 32 ± 0.75C scenarios resulted in 31 extinct replicates before 5 successful replicates were accumulated.

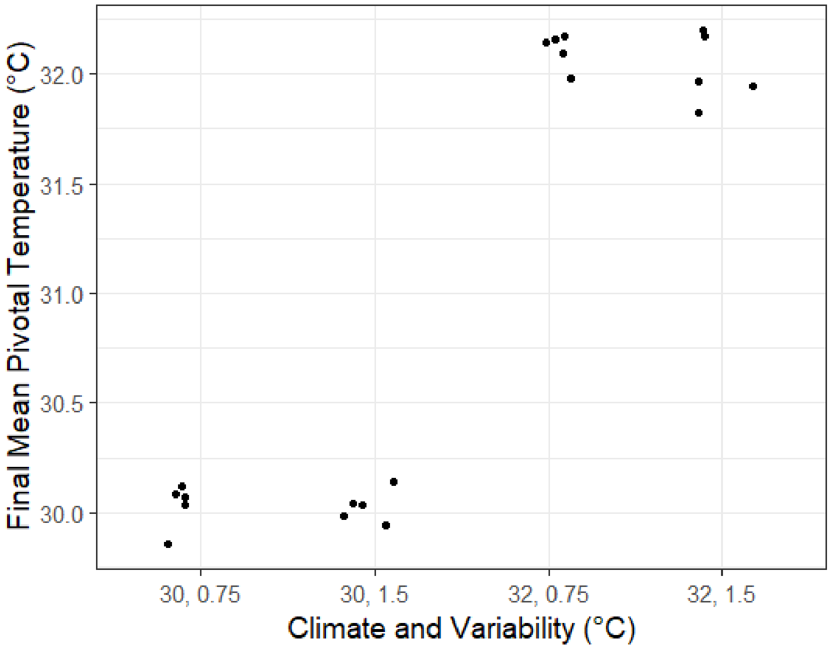

Figure S1. Validation of T_{piv} evolution under two levels of climatic warming (30°C and 32°C) and climate variability (0.75°C and 1.5°C).

Slope Evolution - Validation of selection for ESD

We validated that ESD is selected for under a baseline climate scenario (no climatic warming). T_{piv} shift was set to 0. Neither T_{piv} shift nor T_{piv} could mutate. In these validation replicates, populations with low initial mean slope evolved steeper (more negative) slopes (Fig. S2). When populations were initiated with slopes steeper than -3, their mean slope values generally remained close their starting value. These results suggests that ESD is selected for, but that once the slope is very steep, there is little selection for steeper slopes.

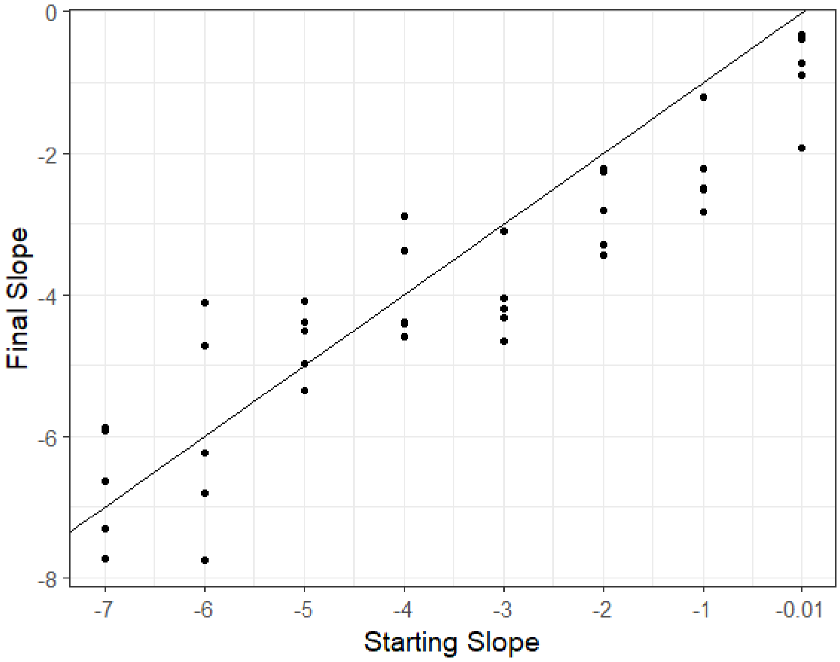

Figure S2. Comparison of final slopes resulting from starting slopes ranging from -0.01 to -7. Scenarios with final slopes steeper than their starting slope fall above the diagonal line, while scenarios with final slopes that are shallower than their starting slope fall below the line. Results are from scenarios with a global temperature of 28°C, climate variability of 0.75°C, and low shift plasticity. Scenarios ran for 50000 iterations. N = 5 for each starting slope value.

T_{piv} Shift Evolution

We validated that shift is selected for in the baseline climate and in the absence of T_{piv} evolution. There was considerable variation in final shift values. In general, scenarios with a starting level of shift at and above 0.4 increased the level of shift over the course of the simulation (Fig. S3). This suggests that shift can evolve if genes for a threshold value of shift exist in the population.

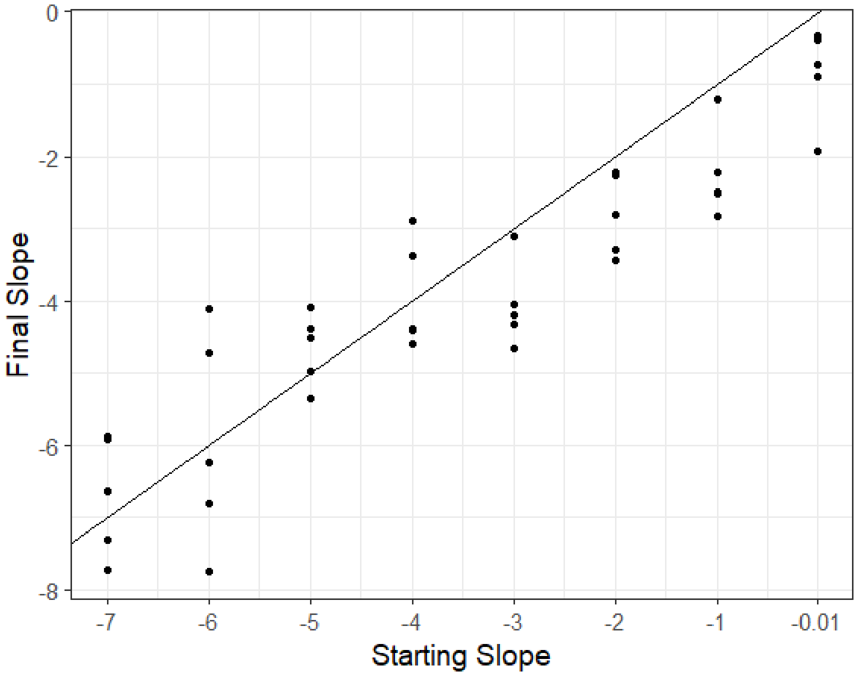

Figure S3. Comparison of final shift values resulting from starting shift values ranging from 0 to 1. Scenarios with final shifts greater than starting shifts fall above the line, while scenarios with final shifts lower than starting shifts fall below the line. Results are from scenarios with a global temperature of 28°C, climate variability of 0.75°C, and high slope plasticity. Scenarios ran for 50000 iterations. N = 5 for each starting shift value.

Sensitivity to Mutation Range

We examined sensitivity of simulation results to mutation by varying the breadth of new mutational values possible for chosen mutants. When the slope of the reaction norm was allowed to evolve, we detected sensitivity to mutation range in both T_{piv} and slope evolution (Fig. S4, Table S3). In low slope plasticity scenarios, broader mutation range was associated with increased evolution of T_{piv} toward the new environmental mean (Fig. S4 first and second row, left). Mutation range did not affect T_{piv} evolution in the high plasticity scenarios. Mutation range affected slope evolution in both the high and low plasticity groups. Steeper slopes were achieved when range was increased (Fig. S4 first and second row, right). In general, as mutation range increased, populations were more likely to retain ESD (steeper slope), and the T_{piv} ended closer to the new climate mean (Table S3). At 3% mutation range populations were less likely to go extinct (Table S3).

In scenarios where shift was able to evolve, there was less sensitivity to mutation range than seen in slope scenarios. Greater mutation range modestly increased the evolvability of T_{piv} in some replicates in the low plasticity scenarios (Fig. S4 third and fourth rows, left). Final shift values were slightly lower in higher mutation scenarios for all run types. Again, this effect was stronger in the low plasticity scenarios (Fig. S4 third and fourth rows, right). In general, as mutation range increased, fewer populations went extinct, populations had lower shifts, and the T_{piv} ended closer to the new climate mean in some of the populations (Table S4).

Overall, it seems that greater mutation range facilitated the directional evolution of T_{piv}, thereby reducing frequency-dependent selection for shallow slopes and large shifts.

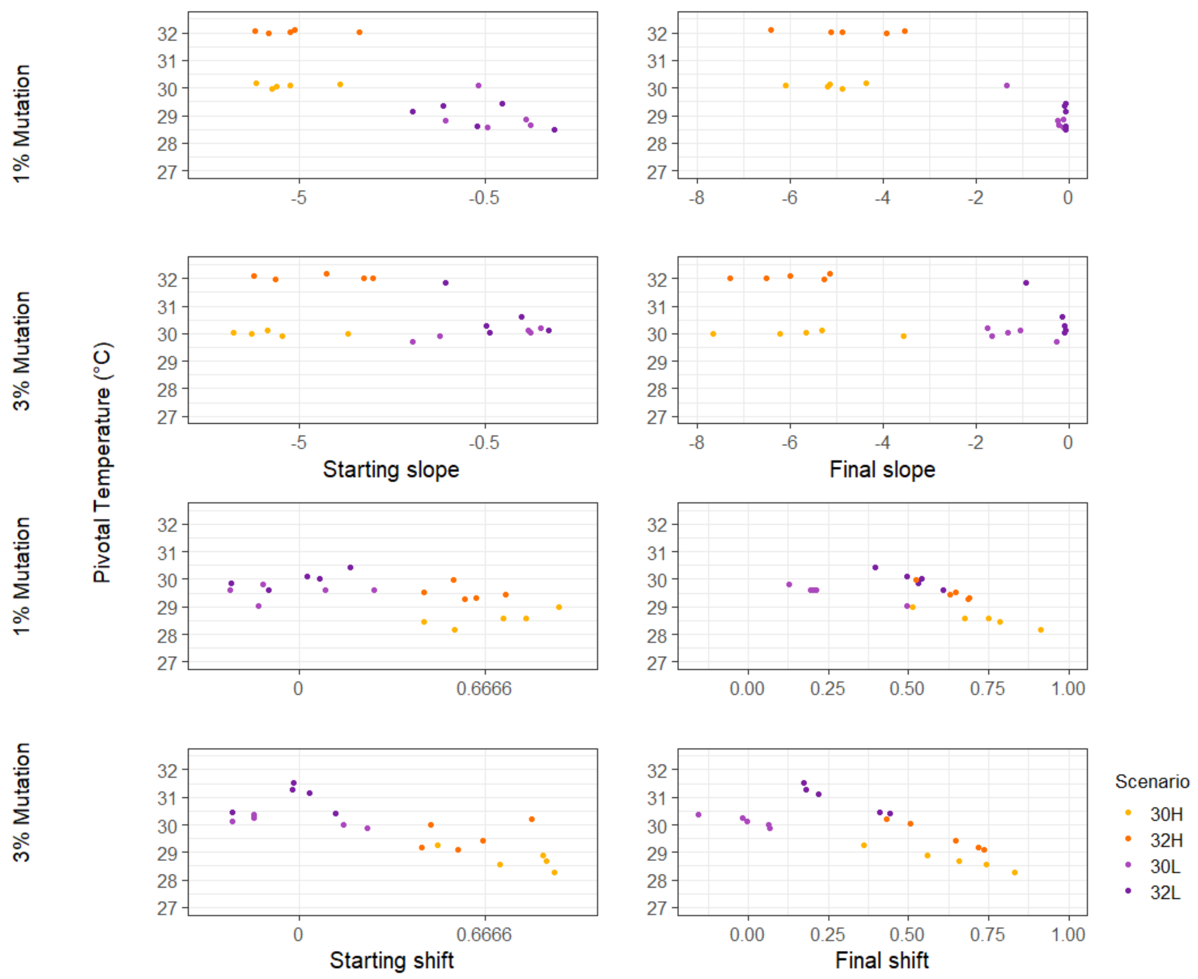

Figure S4. Analysis of the sensitivity of T_{piv}, slope, and shift evolution to two levels of mutation range (1% vs 3% of the trait mean). In the left column, points are jittered around two categorical scenarios on the x-axis.

Table S3. Summary of the sensitivity of T_{piv} and slope evolution to variation of mutation in T_{piv} and slope. The 1% and 3% mutation range results have a sample size of n=5. The 2% mutation range results are presented in the main text and have higher, variable sample sizes. They are presented here again for comparison.

**Mutation Variability**	**Global Temperature**	**Starting Slope**	**Mean Final T_{piv}**	**Mean Final Slope**
1%	30°C	-0.5	29.0°C	-0.411
		-5	30.1°C	-5.183
	32°C	-0.5	29.0°C	-0.068
		-5*	32.1°C	-4.826
2%	*30°C*	*-0.5*	*29.9°C*	*-1.308*
		-5	*30.0°C*	*-5.050*
	32°C	*-0.5*	*29.2°C*	*-0.076*
		-5†	*32.0°C*	*-4.785*
3%	30°C	-0.5	30.1°C	-1.184
		-5	30.0°C	-5.677
	32°C	-0.5	30.6°C	-0.277
		-5‡	32.0°C	-6.008

*26.3% survival (5/19 runs), †16.7% survival (4/24 runs), ‡ 83.3% survival (5/6 runs)

Table S4. Summary of the sensitivity of T_{piv} and shift evolution to variation of mutation in T_{piv} and shift. All analyses were conducted with a sample size of five replicates. The 1% and 3% mutation range results have a sample size of n=5. The 2% mutation range results are presented in the main text and have higher, variable sample sizes but have been provided for comparison.

**Mutation Variability**	**Global Temperature**	**Starting Shift**	**Mean Final T_{piv}**	**Mean Final Shift**
1%	30°C	0	29.5°C	0.254
		0.6666	28.6°C	0.728
	32°C	0*	30.0°C	0.502
		0.6666	29.5°C	0.627
2%	*30°C*	*0*	*29.8°C*	*0.121*
		0.6666	*28.8°C*	*0.654*
	32°C	*0†*	*30.4°C*	*0.411*
		0.6666	*29.3°C*	*0.693*
3%	30°C	0	30.2°C	-0.011
		0.6666	28.8°C	0.627
	32°C	0‡	30.9°C	0.2883
		0.6666	29.6°C	0.602

*23.8% survival (5/21 runs), †25.9% survival (7/27 runs), ‡83.3% survival (5/6 runs)

Sensitivity to Adult Mortality Rate

When simulated individuals had shorter lifespans (0.5% = 2-year life expectancy (second and fourth row) vs. 0.02% = 50-year life expectancy (first and third row)), the starting slope had a reduced effect on pivotal temperature evolution. All short-lived populations experienced evolution of the Tpiv to match the mean climate. In long lived populations, only high slope plasticity was associated with pivotal temperature evolution (Fig. S5, first *vs*. second rows, left, Table S5).

In long-lived populations, starting slope influenced slope evolution. Populations with steeper slopes were more likely to maintain ESD-like slopes than populations with shallow starting slopes (Fig. S5, first row, left). In contrast, starting slope and climate interacted to influence slope evolution populations with short life spans. Specifically, high plasticity 30°C replicates maintained steep slopes, and high plasticity 32°C replicates evolved shallower ESD-like slopes while populations with low plasticity maintained shallow ESD slopes regardless of climate (Fig. S5 second row right panel, Table S3 5).

Increased pivotal temperature evolution and maintenance of ESD in short-lived populations is likely due to strong frequency dependent selection on the pivotal temperature. In long-lived populations shallow slopes are selected for due to the increased chance of producing a rare male. However, as producing a male in a hot climate is very unlikely, shallow slopes are unlikely to benefit an individual that only breeds once or twice. Therefore, pivotal temperature evolution is a more reliable path to producing the rare sex in short-lived populations. The exception is in high slope plasticity populations in hot climates, these scenarios experience quick pivotal temperature evolution to the new mean temperature, and subsequent evolution of a shallower slope. This suggests that while pivotal temperature evolution is the most important factor for adaptation to rising temperatures in short-lived populations, evolving a shallower slope and therefore a greater chance of producing a mixed-sex nest is also advantageous in extreme climates.

Starting shift had less influence on final T_{piv} in populations with shorter lifespans (Fig. S5, third *vs.* fourth rows, right, Table S6). This was because shorter lifespans led to lower final pivotal temperatures and higher shift across every scenario compared to populations with longer lifespans (Fig. S5, fourth row, right, Table S6). In long-lived populations, the extent of climatic warming impacted shift evolution, 32°C scenarios were more likely to evolve greater shift than 30°C scenarios (Fig S5, third row, dark vs light symbols, Table 6). The relationship between climate and shift evolution is similar to the trend we observed in the main simulation (Fig. 4d, main text).

Overall, for short lived populations, shift evolution was a more advantageous strategy than pivotal temperature evolution. This is probably because being able to respond directly to annual climate is especially useful if individuals have a reduced number of breeding seasons and therefore a reduced chance of producing offspring of the rare sex. Similarly, long-lived 32°C populations may have very few years in which producing a male is possible, despite their longevity. Thus, evolution of shift plasticity was favoured in these populations.

Low mortality and plasticity that ameliorated sex ratio bias (high shift, shallow slope) promoted population survival, while high mortality exacerbated population decline (Tables S5&S6). It is important to note that the extinction rate for short-lived populations with steep slopes or low shift plasticity was very high, so these results are from a subset of unusual populations.

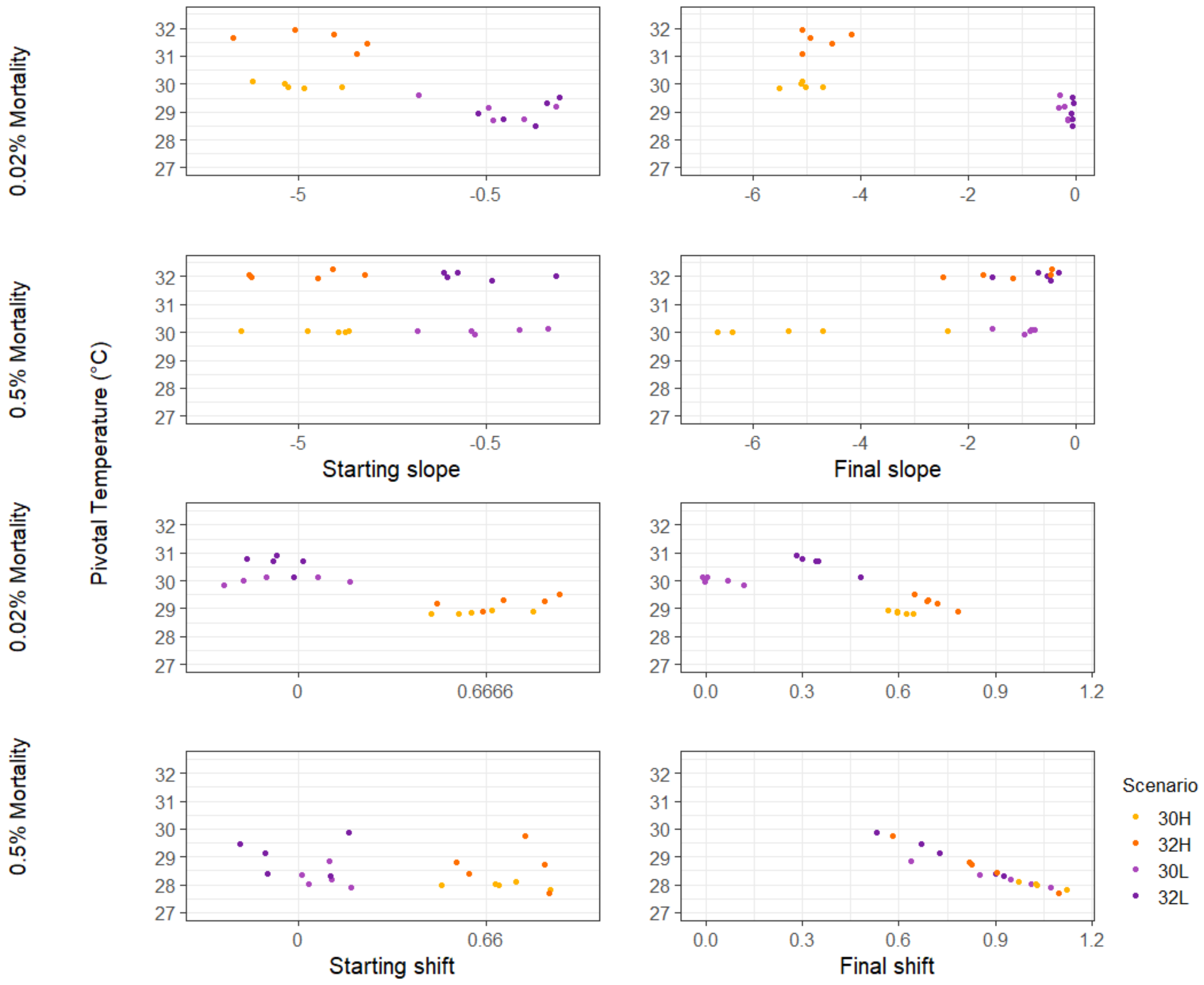

Figure S5. Sensitivity of T_{piv}, slope, and shift evolution to two levels of adult mortality (0.02% and 0.5%).

Table S5. Summary of the sensitivity of T_{piv} and slope evolution to variation in adult mortality rate. The 0.02 and 0.5 mortality rate results have a sample size of n=5. The 0.1 mortality rate results are from the main text and have higher, variable sample sizes but have been provided for comparison.

**Adult Mortality**	**Global Temperature**	**Starting Slope**	**Mean Final T_{piv}**	**Mean Final Slope**
0.02	30°C	-0.5	29.1°C	-0.235
		-5	29.9°C	-5.076
	32°C	-0.5	29.0°C	-0.054
		-5*	31.6°C	-4.755
0.1	*30°C*	*-0.5*	*29.9°C*	*-1.308*
		-5	*30.0°C*	*-5.050*
	32°C	*-0.5*	*29.2°C*	*-0.076*
		-5†	*32.0°C*	*-4.785*
0.5	30°C	-0.5	30.1°C	-1.046
		-5	29.9°C	-5.193
	32°C	-0.5	32.0°C	-0.834
		-5‡	32.0°C	-1.176

*50% survival (5/10 runs), †16.7% survival (4/24 runs), ‡ (5/91 runs)

Table S6. Summary of the sensitivity of T_{piv} and shift evolution to variation in adult mortality rate. The 0.02 and 0.5 mortality rate results have a sample size of n=5. The 0.1 mortality rate results are from the main text and have higher, variable sample sizes but have been provided for comparison

**Adult Mortality**	**Global Temperature**	**Starting Shift**	**Mean Final T_{piv}**	**Mean Final Shift**
0.02	30°C	0	30.0°C	0.034
		0.6666	28.9°C	0.610
	32°C	0*	30.6°C	0.346
		0.6666	29.2°C	0.709
0.1	*30°C*	*0*	*29.8°C*	*0.121*
		0.6666	*28.8°C*	*0.654*
	32°C	*0†*	*30.4°C*	*0.411*
		0.6666	*29.3°C*	*0.693*
0.5	30°C	0	28.2°C	0.934
		0.6666	28.0°C	1.033
	32°C	0‡	29.0°C	0.745
		0.6666	28.6°C	0.843

*38.5% survival (5/13 runs), †25.9% survival (7/27 runs), ‡11.4% survival (5/44 runs)

Results of High Variability (SDbw 1.5°C) Scenarios

The results of scenarios where the standard deviation of inter-annual climate variability was 1.5°C.

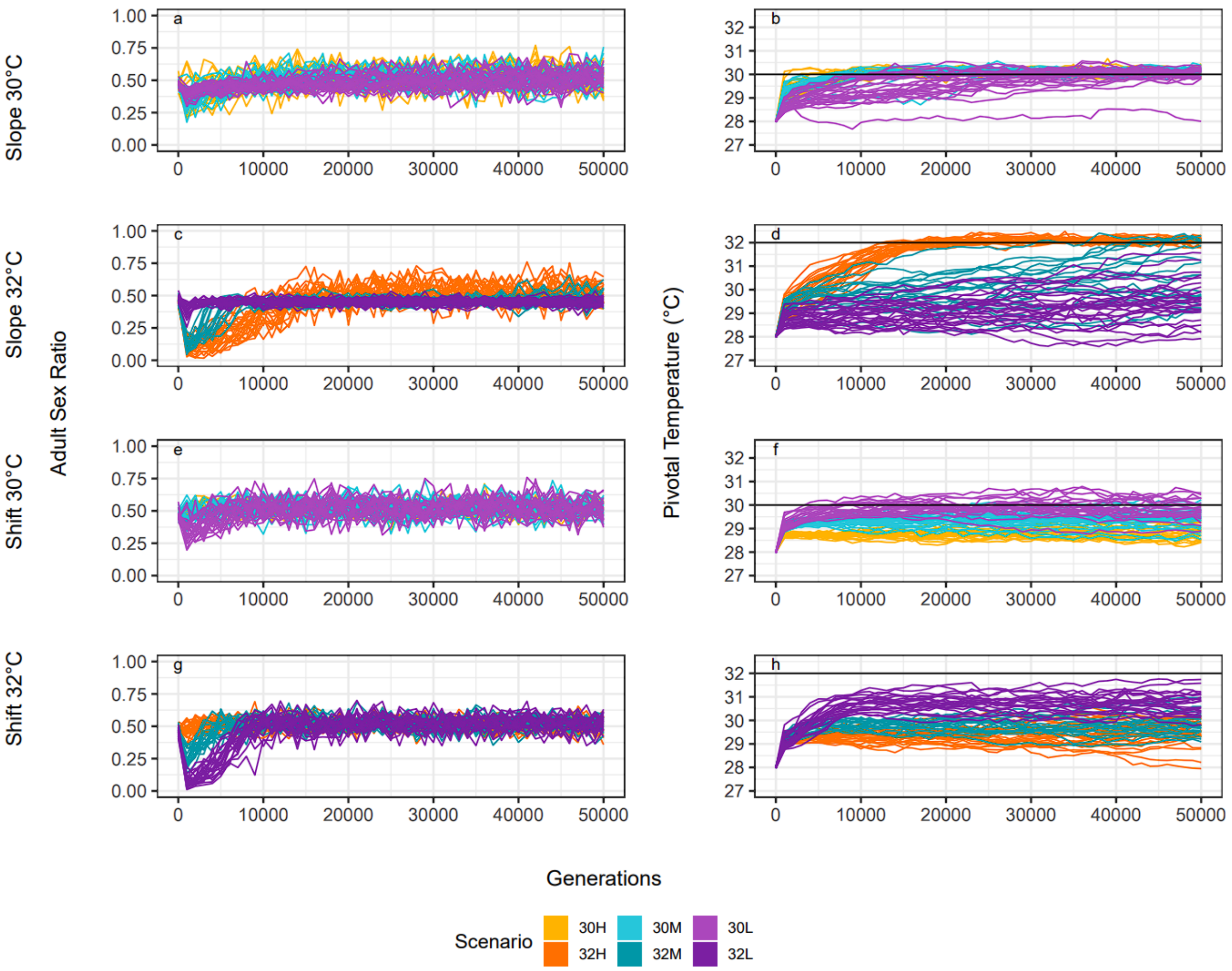

Figure S6. The adult sex ratios and mean pivotal temperatures (T_{piv}) over time for high variability replicate populations at different levels of climate and plasticity. Only results from warmed climate scenarios are presented as there was little change in T_{piv} or adult sex ratio in control climate populations. A ratio of 1 is 100% male, a ratio of 0 is 100% female. Levels of plasticity in the scenario key are: H= High, M= Moderate, L= Low.

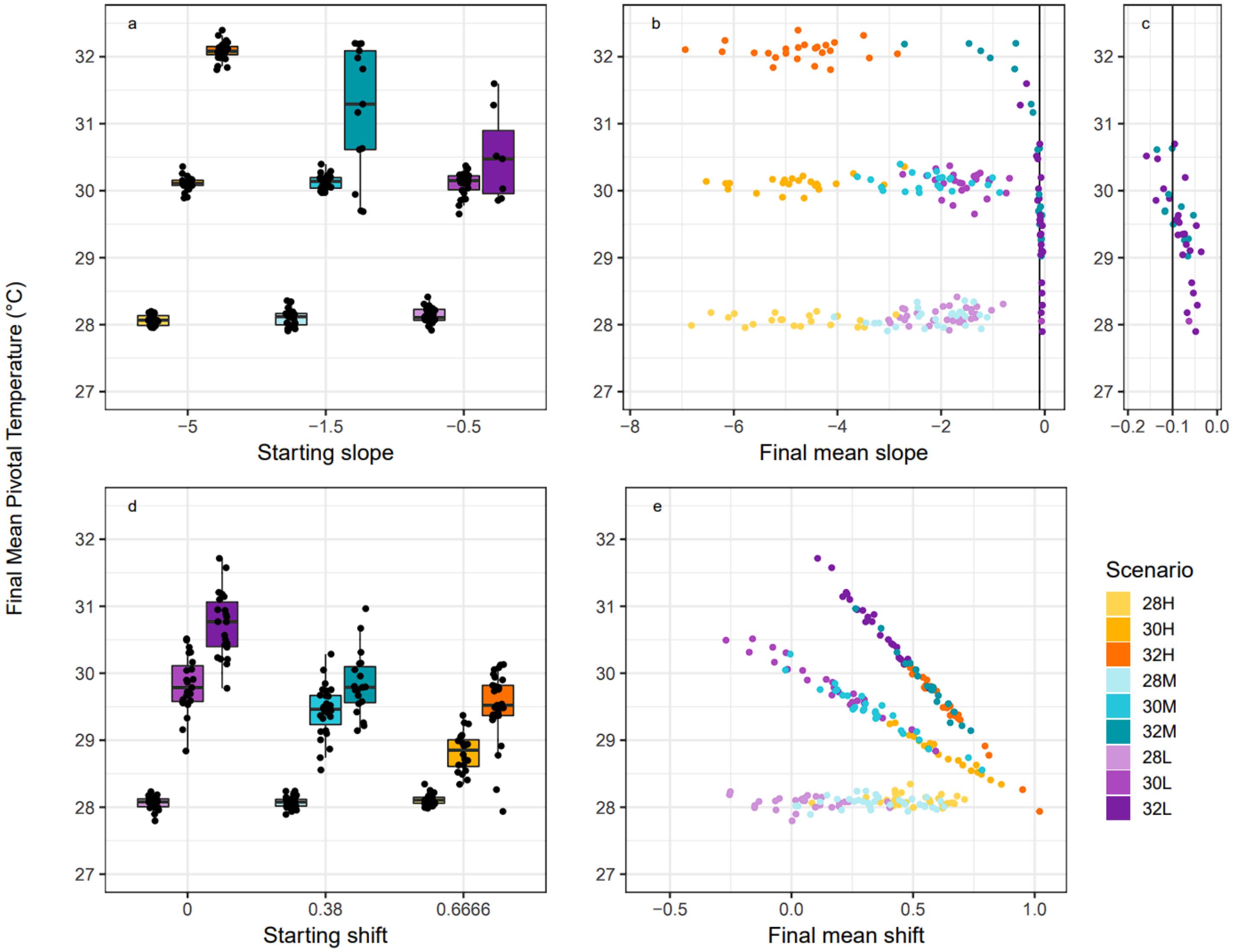

Figure S7. The final mean pivotal temperature of high variability replicate simulations over starting and final mean plasticity levels (mean of the last 1000 generations). Levels of plasticity in the scenario key are: H= High, M= Moderate, L= Low. Results shown are from low climate variability treatments. (a) Final mean pivotal temperatures for the three levels of starting slope plasticity in all climate scenarios. Only replicates that maintained ESD are included in the boxplots. (b) Final mean pivotal temperatures and final mean slopes of replicates in slope plasticity scenarios, the black line indicates the boundary between ESD and a GSD-like slope. (c) An expansion of (b), centered around the black line. It shows the scenarios that lost ESD (slope > -0.1) on the right side of the black line. (d) Final mean pivotal temperatures for the three levels of starting shift plasticity in all climate scenarios. (e) Final mean pivotal temperatures and final mean shifts of replicates in shift plasticity scenarios.
